# Mixed-Metal Oxo Clusters Structurally Derived from Ti_6_O_4_(OR)_8_(OOCR′)_8_

**DOI:** 10.1002/ejic.201402499

**Published:** 2014-08-15

**Authors:** Christine Artner, Ayse Koyun, Matthias Czakler, Ulrich Schubert

**Affiliations:** a Institute of Materials Chemistry, Vienna University of Technology Getreidemarkt 9, 1060 Vienna, Austria

**Keywords:** Cluster compounds, Bimetallic compounds, Titanium, Chain structures, Carboxylate ligands

## Abstract

The mixed-metal oxo clusters FeTi_5_O_4_(O*i*Pr)_4_(OMc)_10_ (OMc = methacrylate), Zn_2_Ti_4_O_4_(O*i*Pr)_2_(OMc)_10_, Cd_4_Ti_2_O_2_(OAc)_2_(OMc)_10_(HO*i*Pr)_2_, [Ca_2_Ti_4_O_4_(OAc)_2_(OMc)_10_]*_n_*, and [Sr_2_Ti_4_O_4_(OMc)_12_(HOMc)_2_]*_n_* were obtained from the reaction of titanium alkoxides with the corresponding metal acetates and methacrylic acid. Their structures are derived from Ti clusters with the composition Ti_6_O_4_(OR)_8_(OOCR′)_8_. The Ca and Sr derivatives consist of chains of condensed clusters.

## Introduction

**Figure 1:**
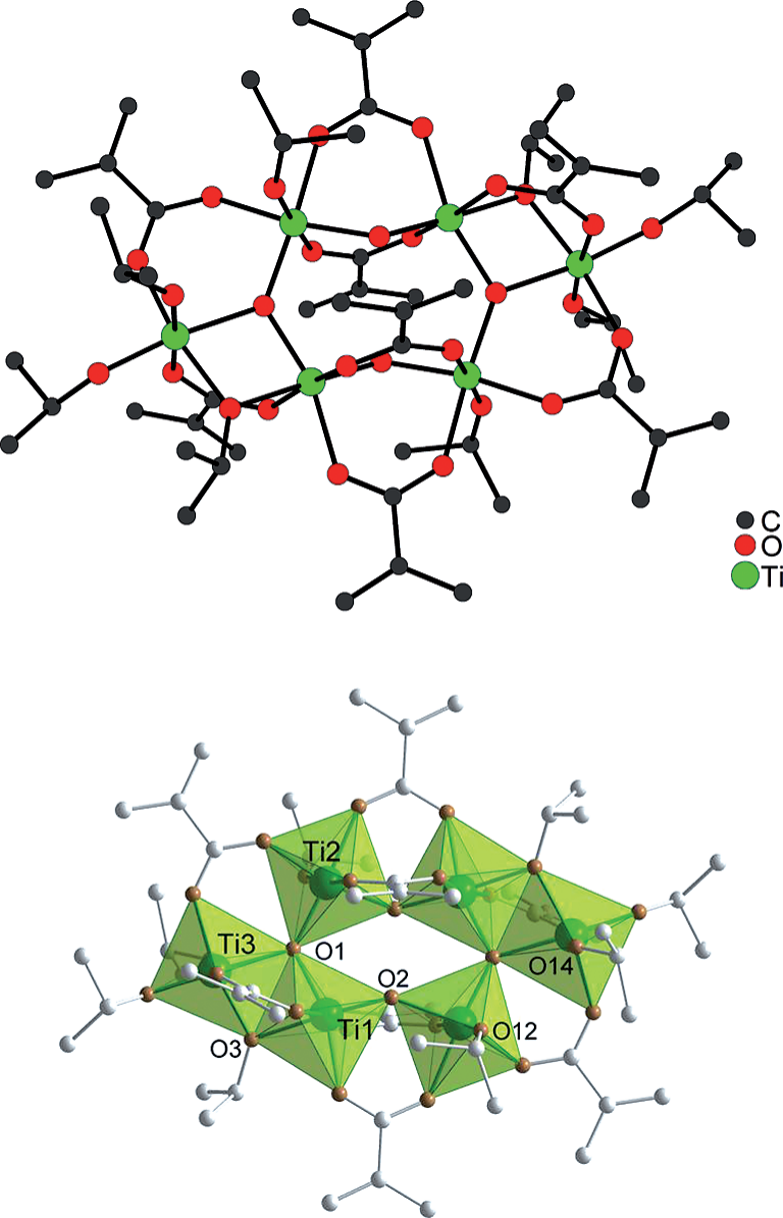
Molecular structure of Ti_6_(μ_3_-O)_2_(μ_2_-O)_2_(μ_2_-O*i*Pr)_2_(μ_2_-OMc)_8_(O*i*Pr)_6_ (Ti6): (top) ball-and-stick model; (bottom) polyhedral representation. Hydrogen atoms are omitted for clarity. Selected bond lengths and angles: Ti1–Ti2 3.3783(3), Ti1–Ti3 3.1062(3), Ti2–Ti3 3.5862(4), Ti1–O1 1.9047(9), Ti1–O2 1.7497(9), Ti1–O3 2.0874(9), Ti1–O4 2.0024(10), Ti1–O6 2.0465(10), Ti1–O8 2.0051(10), Ti2–O2 1.8893(9), Ti2–O1 1.9061(9), Ti2–O5 2.1019(10), Ti2–O7 2.0892(10), Ti2–O10 2.0300(10), Ti2–O12 1.7598(11), Ti3–O1 2.0722(9), Ti3–O3 1.9572(10), Ti3–O9 2.1627(11), Ti3–O11 2.0482(11), Ti3–O13 1.7995(10), Ti3–O14 1.7903(11) Å; Ti1–O1–Ti2 128.00(5), Ti1–O1–Ti3 102.63(4), Ti2–O1–Ti3 128.65(5)°.

Oxo clusters of the general composition Ti*_a_*O*_b_*(OR)*_c_*(OOCR′)*_d_* are obtained when titanium alkoxides, Ti(OR)_4_, are treated with more than one molar equivalent of a carboxylic acid.^1^ The carboxylic acid not only provides carboxylate ligands but also acts as an in situ water source through esterification with the eliminated alcohol. The outcome of the reaction depends, among others, on the groups R and R′ as well as the Ti(OR)_4_/R′COOH ratio. Many oxo clusters have been isolated with different degrees of condensation (*a*:*b* ratio), different degrees of substitution (*a*:*d* ratio), different proportions of residual OR groups (*a*:*c* ratio), and, as a consequence, different structures. One of the more prominent structure types is Ti_6_O_4_(OR)_8_(OOCR′)_8_, which was obtained for several R/R′ combinations.^2^,^3^ An example, with R = *i*Pr and OOCR=OMc (Ti6) (HOMc = methacrylic acid), is shown in Figure1.

In this article, we describe the structures of mixed-metal oxo clusters that are derived from that of Ti_6_O_4_(OR)_8_(OOCR′)_8_. The variability of this structural motif shows that this is a robust structure that not only tolerates variations of R and R′, but also that of the metal polyhedra of which it is composed.

Mixed-metal clusters have been prepared by addition of carboxylic acids to metal alkoxide mixtures.^4^ We have previously shown that use of metal acetates as one of the components is a good alternative, because they are readily available and easy to handle.^5^ The mixed-metal clusters described in this article were therefore prepared from Ti(O*i*Pr)_4_ and metal acetates as precursors [Equation (1)]. We chose a selection of divalent metals (M) with different ionic radii and different preferred coordination numbers to find out how these parameters influence the structures of the clusters, keeping in mind that the charges of the metal as well as the total number of coordination sites must be balanced by the ligands to get a stable cluster.^6^(1)
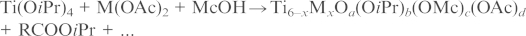


In previous work on carboxylate-substituted metal oxo clusters, we were initially using methacrylic acid to get clusters that can be polymerized subsequently to obtain hybrid materials.^7^ It turned out that methacrylic acid is particularly well suited to the production of crystalline clusters. This may be owing to steric reasons and/or a suitable balance of substitution versus esterification reaction rates. We therefore used methacrylic acid in this work as well, although in this case no subsequent polymerizations were intended.

## Results and Discussion

Although several clusters of the composition Ti_6_O_4_(OR)_8_(OOCR′)_8_ are known with various R/R′ combinations, we prepared the derivative with R = *i*Pr and OOCR=OMc (Ti6) (Figure1) for better comparison of the structural parameters with that of the mixed-metal clusters reported in this article. The centrosymmetric Ti_6_O_4_ core is formed by two Ti_3_(μ_3_-O) units, which are connected through two μ_2_-oxygen atoms. An alternative description of the structure is that of a Ti_4_O_4_ ring of four corner-sharing octahedra to which two Ti octahedra (called “outer Ti” in the following) are condensed through shared edges. Balancing of charges and coordination numbers is achieved by two bridging O*i*Pr ligands (connecting the two edge-sharing octahedra), eight bridging carboxylate ligands and six terminal O*i*Pr ligands.

**Figure 2:**
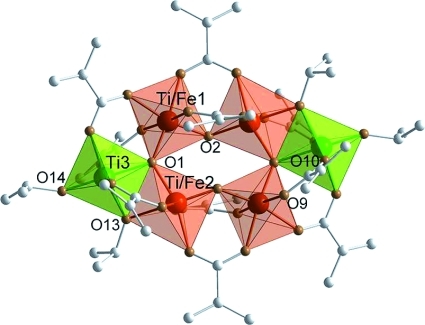
Molecular structure of FeTi_5_(μ_3_-O)_2_(μ_2_-O)_2_(μ_2_-O*i*Pr)_2_(O*i*Pr)_2_(μ_2_-OMc)_10_ (FeTi5). Hydrogen atoms are omitted for clarity. Selected bond lengths and angles: Fe/Ti1–Fe/Ti2 3.4215(10), Fe/Ti1–Ti3 3.4917(10), Fe/Ti2–Ti3 3.0466(11), Fe/Ti1–O1 1.918(3), Fe/Ti1–O2 1.875(3), Fe/Ti2–O1 1.904(3), Fe/Ti2–O2 1.776(3), Ti3–O1 1.990(3), Fe/Ti1–O7 2.044(3), Fe/Ti1–O9 2.040(3), Fe/Ti2–O11 2.030(3), Ti3–O8 2.025(4), Ti3–O10 1.974(4), Ti3–O12 2.031(4), Fe/Ti1–O3 2.015(3), Fe/Ti1–O5 2.061(3), Fe/Ti2–O4 2.012(3), Fe/Ti2–O6 2.047(3), Fe/Ti2–O13 2.137(3), Ti3–O13 1.902(3), Ti3–O14 1.773(3) Å; Fe/Ti1–O1–Fe/Ti2 127.09(15), Fe/Ti1–O1–Ti3 126.61(14), Fe/Ti2–O1–Ti3 102.95(12)°.

Treatment of Fe(OAc)_2_ and Ti(O*i*Pr)_4_ (2 equiv.) with methacrylic acid (17 equiv.) resulted in reddish-brown crystals of FeTi_5_O_4_(O*i*Pr)_4_(OMc)_10_ (FeTi5) (Figure2). The cluster core of FeTi5 is isostructural to that of Ti6. Although attachment of the “outer” Ti octahedra is the same as in Ti6, the four Ti atoms of the Ti_4_O_4_ ring are partly replaced by Fe atoms owing to the nearly identical ionic radii of Ti^4+^ (0.605 Å^8^) and low-spin Fe^2+^ in an octahedral coordination (0.61 Å). Distinction between these two elements in the crystal structure is not straightforward. The Ti/Fe1 site (corresponding to Ti2 in Ti6) was refined with an occupancy for Fe of 34 % and that of Ti/Fe2 (corresponding to Ti1 in Ti6) with 16 %. To prove incorporation of both metals, the crystals were washed with dry *n*-heptane and their metal content was checked with energy-dispersive X-ray spectroscopy (EDX), through which both Fe and Ti were found in the crystals.

FeTi5 needs two negative ligands less than Ti6 because of the lower charge of Fe^2+^, but the total number of coordination sites to be occupied by the ligands is the same because all metal atoms in both Ti6 and FeTi5 are octahedrally coordinated. Thus, the two terminal O*i*Pr groups O12 (on Ti2) and O14 (on Ti3) in Ti6, which are nearly parallel to each other are replaced by one bridging OMc ligand in FeTi5 (O9 and O10) (compare Figures1 and 2). As a consequence of this substitution, the coordination octahedra in FeTi5 are slightly tilted relative to Ti6. This results, among other things, in slightly different distances between the metal centers (3.4215, 3.4917, and 3.0466 Å in FeTi5 compared with 3.3783, 3.5862, and 3.1062 Å in Ti6 for analogous distances).

**Figure 3:**
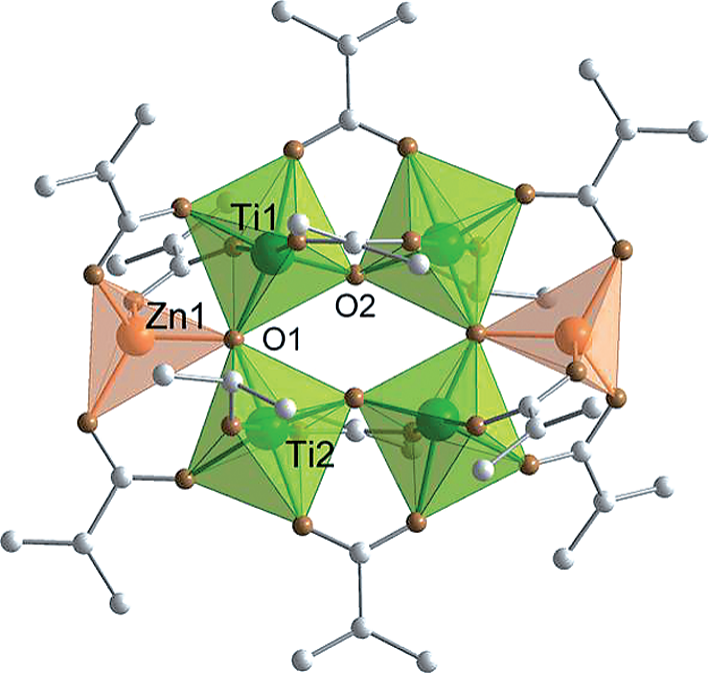
Molecular structure of Zn_2_Ti_4_(μ_3_-O)_2_(μ_2_-O)_2_(O*i*Pr)_2_(μ_2_-OMc)_10_ (Zn2Ti4). Hydrogen atoms are omitted for clarity. Selected bond lengths and angles: Zn1–Ti1 3.1470(4), Zn1–Ti2 3.2701(4), Ti1–Ti2 3.3652(5), Zn1–O1 1.9747(13), Ti1–O1 1.8712(13), Ti1–O2 1.7550(13), Ti2–O1 1.9231(14), Ti2–O2 1.8784(13), Ti2–O(13) 1.7821(14), Zn1–O3 1.946(2), Zn1–O5 1.959(2), Zn1–O7 1.940(2), Ti1–O4 2.106(2), Ti1–O6 2.0123(14), Ti2–O8 2.0435(14), Ti1–O9 2.0752(14), Ti1–O11 1.9994(14), Ti2–O10 2.0383(15), Ti2–O12 2.0936(14) Å; Zn1–O1–Ti1 109.80(7), Zn1–O1–Ti2 114.05(6), Ti1–O1–Ti2 136.01(7)°.

Reaction of Zn(OAc)_2_ with Ti(O*i*Pr)_4_ and methacrylic acid in different molar ratios afforded the centrosymmetric cluster Zn_2_Ti_4_O_4_(O*i*Pr)_2_(OMc)_10_ (Zn2Ti4, Figure3). The structure of this cluster is again structurally related to that of Ti6, with the two outer Ti octahedra being replaced by Zn tetrahedra. Contrary to Ti6 and FeTi5, in which the outer Ti octahedra share an edge with one of the octahedra of the Ti_4_O_4_ unit, the Zn tetrahedra share a corner with two Ti octahedra. The μ_3_-oxygen (O1) is slightly shifted towards the titanium atoms [Zn–O1 1.975(1) Å, Ti–O1 1.871(1) and 1.923(1) Å], which results in a widening of the Ti1–O1–Ti2 angle to 136.01(7)° compared to 128.00(5)° in Ti6 and 127.1(1)° in FeTi5.

Zn2Ti4 needs four negative ligands less than Ti6 (and two less than FeTi5) to compensate the metal charges because two Ti^4+^ are replaced by two Zn^2+^. The total number of coordination sites to be occupied by the ligands is reduced by four relative to Ti6 and FeTi5 (replacement of two octahedra by two tetrahedra). Compared to Ti6, four terminal O*i*Pr ligands are missing (the ones at Ti3 and Ti3* in Ti6), and the O*i*Pr ligands bridging Ti1 and Ti3 in Ti6 are replaced by a bridging OMc ligand in Zn2Ti4.

In addition to the μ_3_-oxygen atom (O1), the Zn atom is connected to both neighboring Ti atoms through bridging OMc ligands, two to Ti1 and one to Ti2. The oxygen atoms of these three OMc ligands, together with the μ_3_-oxygen atom, form a tetrahedron around Zn. The Zn–O distances of the OMc ligands vary only slightly between 1.940(2) and 1.957(2) Å and are not much shorter than the Zn–O1 distance of 1.975(1) Å. The O–Zn–O angles vary between 103.24(6) and 116.79(6)°.

**Figure 4:**
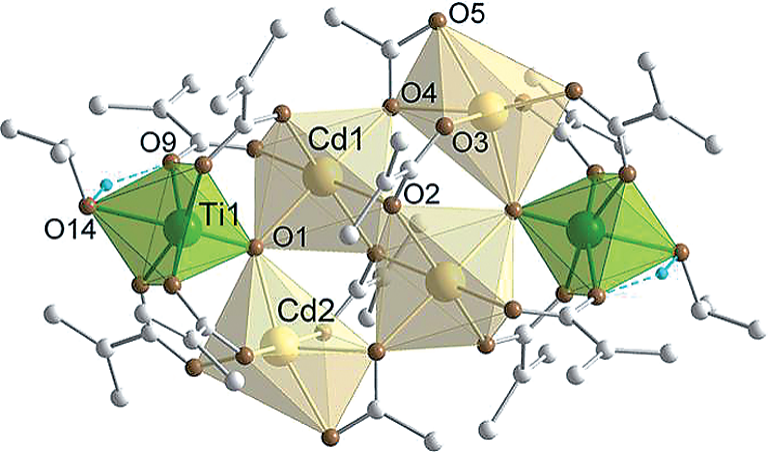
Molecular structure of Cd_4_Ti_2_(μ_3_-O)_2_(μ_3_-OAc)_2_(μ_3_-OMc)_2_(μ_2_-OMc)_8_(HO*i*Pr)_2_ (Cd4Ti2). Only hydrogen of the OH groups are shown. Selected bond lengths and angles: Ti1–Cd1 3.4499(9), Ti1–Cd2 3.4521(9), Cd1–Cd2 3.8904(8), Ti1–O1 1.698(2), Cd1–O1 2.262(2), Cd2–O1 2.267(2), Cd1–O2 2.260(2), Cd1′–O2 2.355(2), Cd1–O4 2.232(2), Cd2–O3 2.267(3), Cd2–O4 2.336(2), Cd2–O5 2.368(2), Ti1–O7 2.012(2), Ti1–O9 1.997(2), Ti1–O11 1.977(2), Ti1–O13 1.986(2), Ti1–O14 2.227(2), Cd1–O6 2.256(2), Cd1–O8 2.260(2), Cd2–O10 2.279(3), Cd2–O12 2.228(3) Å; Cd1–O1–Cd2 118.40(9), Cd1–O1–Ti1 120.55(11), Cd2–O1–Ti1 120.36(11)°.

Reaction of Cd(OAc)_2_ and an equimolar proportion of Ti(O*i*Pr)_4_ with a tenfold excess amount of methacrylic acid resulted in centrosymmetric Cd_4_Ti_2_O_2_(OAc)_2_(OMc)_10_(HO*i*Pr)_2_ (Cd4Ti2, Figure4). The structure of this cluster can again be related to that of Ti6, but there are more profound changes. (i) In contrast to the structures discussed before, the four Ti atoms in the center are replaced by Cd atoms, (ii) acetate groups [originating from the Cd(OAc)_2_ precursor] were incorporated in the structure, (iii) two Cd atoms are bridged across the Cd_4_O_4_ ring, and (iv) the structure contains no μ_2_-oxygen atoms, only the μ_3_-O units are retained.

The titanium atoms are symmetrically connected to the central Cd_4_ unit through four bridging OMc ligands each (two to each neighboring Cd atom) as well as a μ_3_-O atom connecting Cd1, Cd2, and Ti1. Ti1 is approximately equidistant to both Cd [Ti1–Cd1 3.4499(9) Å, Ti1–Cd2 3.4521(9) Å]. The octahedral coordination of Ti1 is completed by a coordinated isopropyl alcohol. The alcoholic proton was identified in the electron density map, and the long Ti1–O14 distance [2.2186(3) Å] proves additionally that this is a coordinated alcohol rather than a O*i*Pr group. The very short Ti–O1 bond length of 1.698(2) Å is due to the coordinated ROH in the *trans* position.

All the metal atoms in both Ti6 and Cd4Ti2 are octahedrally coordinated. The total positive charge of the metals, however, is +24 in Ti6 but only +16 in Cd4Ti2. This means that a smaller number of (monoanionic) ligands must satisfy the coordination requirements of the metals. In addition to the ligands discussed before, coordination of the Cd_4_ core must be completed by two OMc and two OAc ligands. This can only be achieved if each of the carboxylate ligands is tridentate. Thus, one oxygen atom (O2) of the remaining OMc ligands bridges Cd1 and Cd1′, and the second (O3) is coordinated to Cd2. The acetate ligands are bridging-chelating [O4 bridges Cd1 and Cd2; O4 and O5 chelate Cd2]. The coordination octahedron of Cd2 is much more distorted than that of Cd1 due to the chelating carboxylate. Whereas the *cis* O–Cd1–O angles of Cd1 are between 75.0(1) and 97.9(1)°, those of Cd2 are between 55.50(8) and 109.49(9)°.

The μ_3_-OMc and the chelating/bridging acetate cause a shorter distance between the symmetry-related Cd1 atoms than the corresponding Ti atoms in Ti4 or Zn2Ti4. The distance between Cd1 and Cd1′ [3.6618(8) Å] is comparable to that of Cd1–Cd2 [3.8904(8) Å] and Cd1–Cd2′ [3.9525(8) Å]. However, the symmetry-related Cd2 atoms are moved further apart [Cd2–Cd2′ 6.9358(14) Å].

Another carboxylate-substituted Cd/Ti oxo cluster reported in the literature, Cd_4_Ti_4_O_6_(OCHCH_2_NMe_2_)_4_(OCCF_3_)_4_(OAc)_4_,^9^ is also based on four interconnected Cd polyhedra. In this case, however, the Cd_4_ unit is capped by two condensed Ti octahedra on both sides.

**Figure 5:**
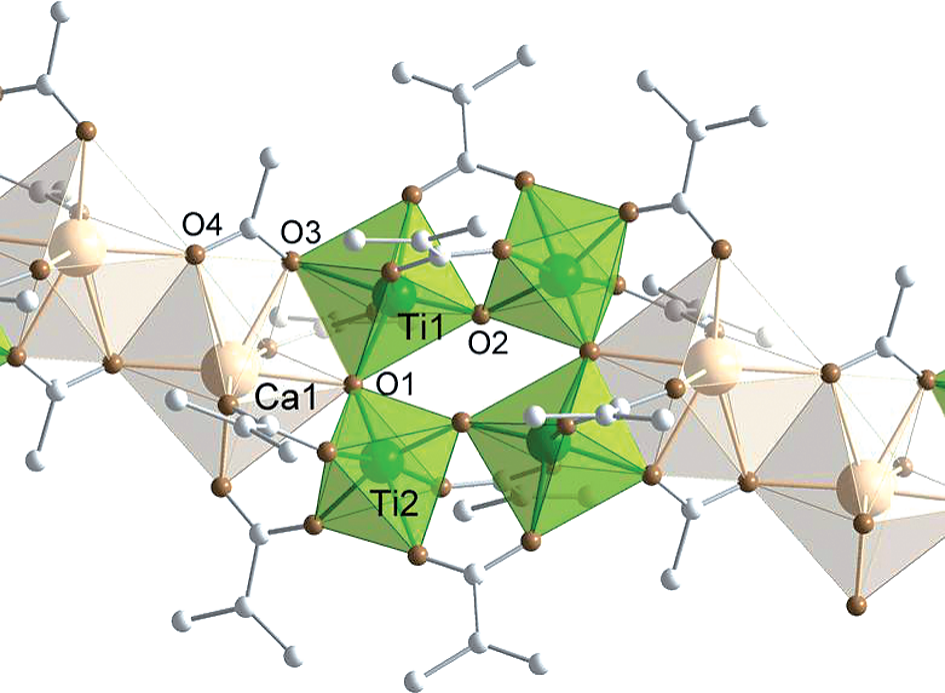
Structure of [Ca_2_Ti_4_(μ_3_-O)_2_(μ_2_-O)_2_(μ_3_-OAc)_2_(μ_2_-OMc)_10_]*_n_* (Ca2Ti4). Hydrogen atoms are omitted for clarity. Selected bond lengths and angles: Ca1–Ca1′ 4.0045(9), Ca1–Ti1 3.5083(6), Ca1–Ti2′ 3.6524(6), Ca1–O1 2.4549(16), Ti1–O1 1.965(2), Ti1–O2 1.730(2), Ti2–O1 1.760(2), Ti2–O2 1.912(2), Ca1–O3 2.428(2), Ca1–O4 2.659(2), Ca1′–O4 2.292(2), Ca1–O5 2.361(2), Ca1–O7 2.317(2), Ca1–O9 2.342(2), Ti1–O3 2.1116(18), Ti1–O6 1.9633(18), Ti2–O8 1.950(2), Ti2–O10 1.958(2), Ti1–O11 2.021(2), Ti1–O13 2.004(2), Ti2–O12 2.026(2), Ti2–O14 2.159(2) Å; Ca1–O1–Ti1 104.55(7), Ca1–O1–Ti2 119.20(8), Ti1–O1–Ti2 135.64(9)°.

Reaction of an equimolar mixture of Ca(OAc)_2_ and Ti(O*i*Pr)_4_ or Ti(OBu)_4_ with methacrylic acid (4.5 equiv.) resulted in crystals of [Ca_2_Ti_4_O_4_(OAc)_2_(OMc)_10_]*_n_* (Ca2Ti4) besides much of a colorless precipitate of unknown composition. In Ca2Ti4 the outer two Ti octahedra of the Ti6 structure are replaced by Ca distorted pentagonal bipyramids, but the structure of the Ti_4_O_4_ core is retained, similar to that in Zn2Ti4. Owing to the lower charge of Ca^2+^ and its higher coordination number, the clusters are condensed to endless parallel chains of Ca_2_Ti_4_ units (Figure5), contrary to the molecular clusters discussed before. The Ca_2_Ti_4_ repeating units are connected through a chelating-bridging acetate ligand. The acetate ligand is chelating Ca1, while one of its oxygen atoms (O3) is bridging Ca1 and Ti1, and the other (O4) Ca1 and Ca1′. The Ca1–O4 bond length [2.660(2) Å] is much longer than that of Ca1′–O4 [2.292(2) Å].

In addition to the chelating-bridging acetate ligand and the μ_3_-oxygen, Ca1 is also connected to Ti1 through a bridging OMc ligand. Two OMc ligands bridge Ca1 and Ti2. The Ca–O bond lengths of the OMc ligands differ only slightly [2.317(2) and 2.361(2) Å], as well as the Ti–O bond lengths of these ligands [1.950(2)–1.963(2) Å].

**Figure 6:**
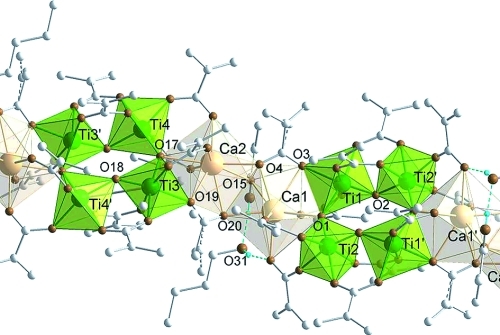
Structure of [Ca_2_Ti_4_(μ_3_-O)_2_(μ_2_-O)_2_(μ_3_-OAc)(μ_3_-OMc)(μ_2_-OMc)_10_(μ_2_-HOMc)·BuOH]*_n_* (Ca2Ti4a). Only hydrogen atoms coordinated to oxygen atoms are displayed. Hydrogen bonds are drawn with blue dashed lines. Grey dashed lines indicate disordered parts. Selected bond lengths and angles: Ca1–Ca2 3.7089(8), Ca1–Ti1 3.5606(7), Ca1–Ti2′ 3.6737(7), Ca1–O1 2.500(2), Ti1–O1 1.931(2), Ti1–O2 1.745(2), Ti2–O1 1.774(2), Ti2–O2 1.875(2), Ca1–O3 2.560(2), Ca1–O4 2.510(2), Ca1′–O4 2.360(2), Ti1–O3 2.101(2), Ca1–O5 2.362(2), Ca1–O7 2.417(2), Ca1–O9 2.358(2), Ti1–O6 1.935(2), Ti2–O8 1.967(2), Ti2–O10 1.957(2), Ca1–O15 2.489(2), Ca2–O15 2.812(2), Ti1–O11 2.025(2), Ti1–O13 2.034(2), Ti2–O12 2.045(2), Ti2–O14 2.127(2) Å; Ca1–O1–Ti1 106.22(8), Ca1–O1–Ti2 117.52(9), Ti1–O1–Ti2 136.12(10)°.

When the molar ratio of Ti(O*i*Pr)_4_/Ca(OAc)_2_ in the precursor mixture was increased from 1:1 to 2:1, while keeping the proportion of methacrylic acid per metal constant, a variation of the structure of Ca2Ti4 was observed. The ligand sphere and the linkage of the cluster units in [Ca_2_Ti_4_O_4_(OAc)(OMc)_11_(HOMc)**·**BuOH]*_n_* (Ca2Ti4a) is slightly different to that of Ca2Ti4, and the repeating cluster unit is doubled. Reasons for this doubling are differences in the occupancy of the ligands. The acetate ligand linking the cluster units is partly substituted by an OMc ligand. The occupancy of the acetate on one site is 25 %, and 75 % on the second (Figure6, indicated by grey dashed lines).

Different to Ca2Ti4, the structure of Ca2Ti4a contains a neutral HOMc ligand, of which one of the oxygen atoms (O15) is unsymmetrically bridging Ca1 and Ca2 [Ca2–O15 2.812(2) Å, Ca1–O15 2.489(2) Å]. This increases the coordination number of Ca from seven in Ca2Ti4 to eight in Ca2Ti4a. The proton of the methacrylic acid is hydrogen bonded to a non-coordinated butanol, and the proton of the butanol in turn to an oxygen atom (O7) of one of the bridging OMc ligands. The bridging HOMc moves the Ca atoms closer to each other [Ca1–Ca2 3.7089(8) compared with 4.0045(9) Å in Ca2Ti4] and also affects the linking carboxylate group. The Ca–O bond lengths of the chelating ligands at Ca1 are now equal [Ca1–O3 2.560(2), Ca1–O4 2.510(2) Å], and the Ca2–O4 distance is slightly lengthened [2.3604(19) Å]. The Ca–O bonds of the chelating carboxylate group at Ca2 differ in lengths, but are still shorter than in Ca2Ti4 [Ca2–O19 2.432(2), Ca2–O20 2.546(2) Å]. All other bond lengths are comparable to those in Ca2Ti4.

**Figure 7:**
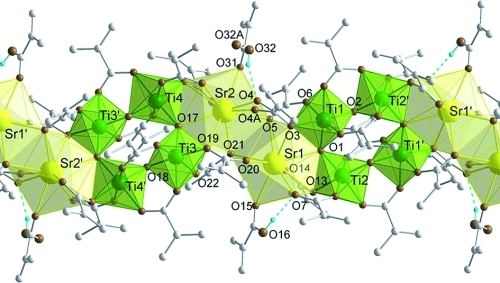
Structure of [Sr_2_Ti_4_(μ_3_-O)_2_(μ_2_-O)_2_(μ_3_-OMc)_6_(μ_2_-OMc)_6_(μ_2_-HOMc)_2_]*_n_* (Sr2Ti4). Only hydrogen atoms coordinated to oxygen atoms are displayed. The blue dashed lines indicate hydrogen bridges. Selected bond lengths and angles: Sr1–Ti1 3.6312(4), Sr1–Ti2′ 3.7280(4), Ti1–Ti2 3.3691(5), Sr1–O1 2.581(2), Ti1–O1 1.803(2), Ti1–O2 1.836(2), Ti2–O1 1.884(2), Ti2–O2 1.777(2), Sr1–O3 2.877(2), Sr2–O4 2.598(4), Sr2–O4A 2.438(13), Sr1–O5 2.595(2), Sr2–O5 2.705(2), Sr1–O7 2.632(2), Sr1–O13 2.946(2), Sr1–O14 2.558(2), Sr1–O15 2.558(2), Ti1–O3 2.017(2), Ti1–O6 1.962(2), Ti1–O9 2.048(2), Ti1–O11 2.087(2), Ti2–O8 1.953(2), Ti2–O13 2.026(2), Ti2–O10 2.013(2), Ti2–O12 2.071(2) Å; Sr1–O1–Ti1 110.59(7), Sr1–O1–Ti2 112.29(7), Ti1–O1–Ti2 137.04(9)°.

We have previously prepared Sr_2_Ti_8_O_8_(O*i*Pr)_1.73_(OAc)_2.27_(OMc)_16_, with a crown ether like structure, from Sr(OAc)_2_, Ti(O*i*Pr)_4_, and methacrylic acid.^5^ A compound with a different structure was obtained when Sr(OAc)_2_ was treated with Ti(OBu)_4_. The arrangement of the metal polyhedra of polymeric [Sr_2_Ti_4_O_4_(OMc)_12_(HOMc)_2_]*_n_* (Sr2Ti4, Figure7) is the same as that of Ca2Ti4 and Ca2Ti4a, but there are differences in the ligand sphere, especially in the coordination of Sr. In addition to μ_3_-oxygen atoms, Sr1 is connected to Ti2 and Sr2 to Ti4 through two bridging OMc ligands each, similar to Ca1 and Ti1 in Ca2Ti4. The connection of Sr1 and Sr2 to Ti3 and Ti1 by two OMc ligands each is slightly different. In one pair of OMc ligands, one oxygen atom of the COO group bridges Sr1 and Ti1, whereas the other oxygen atom coordinates to Sr2 (correspondingly, one oxygen atom bridges Sr2 and Ti3, and the other coordinates to Sr1). In the second pair of OMc ligands, one oxygen atom of the COO group bridges the two Sr atoms and the other oxygen atom binds to Ti1 (or Ti3, respectively).

The coordination spheres of the Sr atoms are completed by an η^1^-HOMc. The coordination number for Sr is nine with two very long Sr–O bonds (>2.8 Å). The methacrylic acid bonded to Sr1 shows hydrogen bonds to the OMc ligand bridging Sr1 and Ti2 [Sr1–O15 2.558(2) Å, O16**···**O7 2.728(2) Å] and that bonded to Sr2 hydrogen bonds to O4 of a μ_3_-OMc ligand [Sr2–O31 2.556(2), O32**···**O4 2.804(5) Å]. The acid as well as O4 are disordered with 75:25 % occupancy of both positions. The minority position shows no hydrogen bonding [O32A**···**O(4A) 3.66(2) Å].

## Conclusion

The structure of all the clusters described in this article can be derived from that of Ti6. The general structure is retained when part of the Ti atoms is replaced by two-valent atoms (Fe^2+^, Zn^2+^, Cd^2+^, Ca^2+^, Sr^2+^), but the lower charge of the second metal renders modification of the ligand sphere necessary. Depending on the preferred coordination number of the two-valent atoms, this adaptation occurs differently. In addition to different coordination of the negatively charged ligands, completion of the coordination sphere is also possible by coordination of neutral ligands (ROH, McOH), as observed in Cd4Ti2, Ca2Ti4a, or Sr2Ti4.

Fe^2+^ and Ti^4+^ have similar bonding characteristics with oxygen and the ionic radii are almost equal (0.61 and 0.605 Å), hence the four inner Ti^4+^ sites are partly replaced by Fe^2+^ atoms (FeTi5). In contrast, the ionic radius of Cd^2+^ is much bigger (0.95 Å). This results in a different arrangement of the coordination octahedra in Cd4Ti2, namely, replacement of the inner Ti atoms by Cd. The lower charge is compensated by a different coordination behavior of the smaller number of ligands.

Although the size of Zn^2+^ is the same as that of Fe^2+^ and Ti^4+^ (0.61 Å), it usually exhibits tetrahedral coordination. Hence a partial substitution of the Ti atoms is not possible. In Zn2Ti4, the two outer Ti octahedra are replaced by Zn tetrahedra, with corresponding adjustment of the connecting ligands.

Ca^2+^ and Sr^2+^ ions are much bigger, have higher coordination numbers (seven-coordinated Ca^2+^ 2.00 Å, eight-coordinate Ca^2+^ 2.17 Å, ten-coordinated Sr^2+^ 2.33 Å), and the bonds are less directed. In the Ca and Sr compounds, the outer Ti octahedra are substituted with Ca or Sr polyhedra. Different to FeTi5, Zn2Ti4, and Cd4Ti2 where molecular clusters were obtained, Ca2Ti4, Ca2Ti4a, and Sr2Ti4 form chains of condensed clusters in the crystal lattice. This is enabled by the higher coordination of Ca^2+^ and Sr^2+^.

The structures reported in this article impressively demonstrate the subtle balancing of charges and coordination behavior of both the metals and the ligands to reach a stable cluster.^6^ In light of the reported results, it is to some extent surprising that such closely related structures were obtained with a given set of ligands, despite the considerably different ionic radii and coordination numbers of the metals.

## Experimental Section

**General:** All experiments were carried out under an argon atmosphere using standard Schlenk techniques. Ti(OBu)_4_, Fe(OAc)_2_, and Sr(OAc)_2_ were obtained from Aldrich, Ti(O*i*Pr)_4_ from ABCR, Ca(OAc)_2_**·**H_2_O and Zn(OAc)_2_**·**H_2_O from Fluka, and Cd(OAc)_2_**·**H_2_O from Merck. Water-free metal acetates were obtained by drying under vacuum at 130 °C overnight (verified by IR spectroscopy). All solvents used for NMR spectroscopy (Eurisotop) were degassed prior to use and stored over molecular sieves. Ti6 was prepared analogously to the O*n*Pr derivative.^2^

^1^H and ^13^C solution-state NMR spectra were recorded on a Bruker Avance 250 (250.13 MHz [^1^H], 62.86 MHz [^13^C]) equipped with a 5 mm inverse-broadband probe head and a *z*-gradient unit.

**General Preparative Procedure:** Ti(O*i*Pr)_4_, the corresponding water-free metal acetate and an excess amount of methacrylic acid were mixed. No solvent was added unless otherwise stated. The mixture was left standing in a closed vessel until crystals were formed.

**FeTi_5_O_4_(O*i*Pr)_4_(OMc)_10_ (FeTi6):** The red-brown solution of Fe(OAc)_2_ (1 mmol, 0.174 g), Ti(O*i*Pr)_4_ (2 mmol, 0.568 g), and methacrylic acid (17 mmol, 1.44 g) was stirred for one week and then filtered. Reddish-brown crystals were obtained after two weeks, yield 0.120 g (41 % rel. Ti).

**Zn_2_Ti_4_O_4_(O*i*Pr)_2_(OMc)_10_ (Zn2Ti4):** Ti(O*i*Pr)_4_ (1 mmol, 0.284 g), dry Zn(OAc)_2_ (1 mmol, 0.183 g), and methacrylic acid (10 mmol, 0.861 g) were mixed at room temperature. After addition of methacrylic acid the mixture immediately turned orange. Dry CH_2_Cl_2_ (1.5 mL) was added to the mixture. After 1 week orange crystals were isolated from the mother liquid, yield (after washing with dry *n*-heptane): 0.256 g (77 % rel. to Ti). ^1^H NMR (CDCl_3_, 250 MHz): *δ* = 1.22 (m, 12 H, CH*Me*), 1.81–2.11 (m, 30 H, =C*Me*), 4.52 (sep, 2 H, C*H*Me), 5.35–5.68 (m, 10 H, =CH_2_), 5.98–6.43 (m, 10 H, =CH_2_) ppm. IR: $\tilde {\nu}$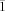 = 611 (s), 686 (w), 790 (m), 825 (m), 859 (w), 942 (m), 988 (w), 1005 (w), 1113 (w), 1162 (w), 1241 (m), 1372 (m), 1411 (s), 1552 (s), 1644 (w), 2975 (w) cm^–1^.

**Cd_4_Ti_2_O_2_(OAc)_2_(OMc)_10_(*i*PrOH)_2_ (Cd4Ti2):** Cd(OAc)_2_ (1 mmol, 0.231 g) was mixed with Ti(O*i*Pr)_4_ (1 mmol, 0.284 g) and methacrylic acid (10 mmol, 0.861 g) in dry CH_2_Cl_2_ (2 mL). The sample was stirred for 3 h and then filtered through a syringe filter. After 2 weeks, colorless crystals could be isolated from the mother liquid, yield (after washing with dry *n*-heptane): 0.198 g (48 % rel. Cd). ^1^H NMR ([D_8_]toluene, 250 MHz): *δ* = 1.36 (m, 12 H, HOCH*Me*_2_), 1.68–1.78 (m, 30 H, =C*Me*), 1.92–2.01 (sep, 6 H, C*Me*), 4.94 (m, 2 H, HOC*H*Me), 5.16–5.40 (m, 10 H, =CH_2_), 6.03–6.45 (m, 10 H, =CH_2_) ppm.

**[Ca_2_Ti_4_O_4_(OAc)_2_(OMc)_10_]*_n_* (Ca2Ti4):** Dry Ca(OAc)_2_ (2 mmol, 0.363 g) and Ti(O*i*Pr)_4_ (2 mmol, 0.568 g) were treated with methacrylic acid (18 mmol, 1.55 g). A clear solution was obtained after 1 h of stirring. After two weeks colorless crystals formed, in addition to much of a white insoluble and amorphous precipitate. The same compound was obtained when dry Ca(OAc)_2_ (2 mmol, 0.363 g) and Ti(OBu)_4_ (2 mmol, 0.702 g) were treated with methacrylic acid (18 mmol, 1.55 g). Colorless crystals were obtained after filtration of the reaction solution.

**[Ca_2_Ti_4_O_4_(OAc)(OMc)_11_(HOMc)·BuOH]*_n_* (Ca2Ti4a):** Dry Ca(OAc)_2_ (1 mmol, 0.182 g) and Ti(O*i*Pr)_4_ (2 mmol, 0.568 g) were treated with methacrylic acid (13.5 mmol, 1.162 g). After two weeks colorless crystals formed, in addition to much of a white insoluble and amorphous precipitate.

**[Sr_2_Ti_4_O_4_(OMc)_12_(HOMc)_2_]*_n_* (Sr2Ti4):** Sr(OAc)_2_ (1 mmol, 0.411 g) and Ti(OBu)_4_ (1 mmol, 0.351 g) were treated with methacrylic acid (12 mmol, 1.03 g). Small amounts of precipitate were formed after three days in the originally clear solution. After 6 weeks a big colorless crystal was formed.

**X-ray Structure Analyses:** Crystallographic data were collected on a Bruker AXS SMART APEX II four-circle diffractometer with κ-geometry at 100 K using Mo-*K*_α_ (*λ* = 0.71073 Å) radiation. The data were corrected for polarization and Lorentz effects, and an empirical absorption correction (SADABS) was employed. The cell dimensions were refined with all-unique reflections. SAINT PLUS software (Bruker Analytical X-ray Instruments, 2007) was used to integrate the frames. Symmetry was then checked with the program PLATON.^10^

The structures were solved by charge flipping (JANA2006). Refinement was performed by the full-matrix least-squares method based on *F*^2^ (SHELXL97) with anisotropic thermal parameters for all non-hydrogen atoms. Hydrogen atoms were inserted in calculated positions and refined riding with the corresponding atom. Crystal data, data collection parameters, and refinement details are listed in Tables 1 and 2.

**Table 1:** Crystal data, data collection parameters, and refinement details.[a]

	Ti6	FeTi5	Zn2Ti4	Cd4Ti2
Empirical formula	C_56_H_96_O_28_Ti_6_	C_26_H_39_Fe_0.5_O_14_Ti_2.5_	C_46_H_64_O_26_Ti_4_Zn_2_	C_50_H_70_Cd_4_O_28_Ti_2_
*M*_r_	1504.73	723.25	1327.43	1664.46
Crystal system	monoclinic	monoclinic	triclinic	monoclinic
Space group	*P*2_1_/*c*	*P*2_1_/*n*	*P*$\bar {1}$ 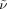	*C*2/*c*
*a* [Å]	11.5503(4)	12.0904(10)	10.0269(3)	22.290(4)
*b* [Å]	18.9581(5)	11.1928(9)	12.7249(4)	15.173(3)
*c* [Å]	17.0246(5)	24.9189(19)	13.1321(5)	21.026(4)
*α* [°]	90	90	106.170 (2)	90
*β* [°]	105.1810(10)	97.830(3)	96.950(2)	110.224(5)
*γ* [°]	90	90	111.950(2)	90
*V* [Å^3^]	3597.81(19)	3340.7(5)	1444.16(8)	6673(2)
*Z*	2	4	1	4
*D*_calcd._ [g* *cm^–3^]	1.389	1.438	1.558	1.379
*μ* [mm^–1^]	0.710	0.859	1.425	0.661
Crystal size [mm]	0.40 × 0.25 × 0.20	0.46 × 0.39 × 0.19	0.19 × 0.18 × 0.15	0.22 × 0.20 × 0.14
*T*_min_, *T*_max_	0.7643, 0.8710	0.6934, 0.8538	0.6213, 0.7473	0.7258, 0.8115
Measd., indep., obsd. reflections [*I* > 2*σ*(*I*)]	103239, 14090, 10638	95122, 8702, 6080	18055, 18055, 13178	122456, 10180, 8084
*R*_int_	0.0523	0.0439	0.0817	0.031
*θ*_max_ [°]	33.50	28.85	37.02	30.52
*R*[*F*^2^ > 2*σ*(*F*)], *ωR*(*F^2^*), *S*	0.0361, 0.889, 1.032	0.0721, 0.1880, 1.119	0.0476, 0.1160, 1.000	0.0367, 0.0839, 1.224
Parameters	418	399	359	391
Weighting scheme[a]	*x* = 0.0459, *y* = 1.6781	*x* = 0.0580, *y* = 11.3336	*x* = 0.0464, *y* = 0.4048	*x* = 0.0111, *y* = 45.3277
*δρ*_max_*, δρ*_min_ [e Å^–3^]	0.955, –0.592	1.065, –0.794	0.918, –0.671	1.534, –0.786

[a] *ω* = 1/[*σ*^2^(*F*_o_^2^) + (*xP*)^2^ + *yP*], in which *P* = (*F*_o_^2^ + 2*F*_c_^2^)/3.

**Table 2:** Crystal data, data collection parameters, and refinement details.[a]

	Ca2Ti4	Ca2Ti4a	Sr2Ti4
Empirical formula	C_22_H_28_CaO_14_Ti_2_	C_54_H_74_Ca_2_O_31_Ti_4_	C_56_H_72_O_32_Sr_2_Ti_4_
*M*_r_	652.32	1490.89	1623.98
Crystal system	orthorhombic	triclinic	triclinic
Space group	*Pccn*	*P*$\bar {1}$ 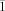	*P*$\bar {1}$ 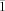
*a* [Å]	20.4104(7)	13.9656(6)	12.7300(4)
*b* [Å]	23.1828(9)	15.3582(7)	13.4769(4)
*c* [Å]	12.5984(4)	16.7388(7)	22.8051(5)
*α* [°]	90	81.340(2)	83.9400(11)
*β* [°]	90	83.406(2)	80.5600(11)
*γ* [°]	90	75.256(2)	63.5300(11)
*V* [Å^3^]	5961.2(4)	3421.4(3)	3452.6(2)
*Z*	8	2	2
*D*_calcd._ [g cm^–3^]	1.454	1.447	1.562
*μ* [mm^–1^]	0.77	0.684	2.06
Crystal size [mm]	0.45 × 0.40 × 0.22	0.29 × 0.23 × 0.19	0.20 × 0.18 × 0.15
*T*_min_, *T*_max_	0.6321, 0.7472	0.6636, 0.7455	0.5171, 0.7465
Measd., indep., obsd. reflections [*I* > 2*σ*(*I*)]	257146, 14564, 9971	101946, 15413, 10380	117417, 21081, 14895
*R*_int_	0.0692	0.071	0.0598
*θ*_max_ [°]	36.74	27.44	30.58
*R*[*F*^2^ > 2*σ*(*F*)], *ωR*(*F^2^*), *S*	0.0645, 0.1799, 1.183	0.0420, 0.1208, 1.062	0.0388, 0.885, 1.020
Parameters	358	850	877
Weighting scheme[a]	*x* = 0.0517, *y* = 13.5390	*x* = 0.0564, *y* = 1.8169	*x* = 0.0344, *y* = 2.5320
*δρ*_max_*, δρ*_min_ [e Å^–3^]	1.724, –0.632	0.829, –0.547	0.844, –0.694

[a] *ω* = 1/[*σ*^2^(*F*_o_^2^) + (*xP*)^2^ + *yP*], in which *P* = (*F*_o_^2^ + 2*F*_c_^2^)/3.

CCDC-1005662 (for Ti6), -CCDC-1005663 (for FeTi5), -CCDC-1005664 (for Zn2Ti4), -CCDC-1005665 (for Cd4Ti2), -CCDC-1005666 (for Ca2Ti4), -CCDC-1005667 (for Ca2Ti4a), and -CCDC-1005668 (for Sr2Ti4) contain the supplementary crystallographic data for this paper. These data can be obtained free of charge from The Cambridge Crystallographic Data Centre via www.ccdc.cam.ac.uk/data_request/cif.
